# Collateral benefits: how the practical application of Good Participatory Practice can strengthen HIV research in sub‐Saharan Africa

**DOI:** 10.1002/jia2.25175

**Published:** 2018-10-18

**Authors:** Deborah Baron, Thandekile Essien, Sinazo Pato, Miliswa Magongo, Nomthandazo Mbandazayo, Fiona Scorgie, Helen Rees, Sinead Delany‐Moretlwe

**Affiliations:** ^1^ Wits RHI University of the Witwatersrand Johannesburg South Africa; ^2^ Department of Health Behavior at the UNC Gillings School of Global Public Health Johannesburg South Africa; ^3^ International Partnership for Microbicides Johannesburg South Africa

**Keywords:** GPP, stakeholder engagement, HIV, prevention research, Africa

## Abstract

**Introduction:**

The *Good Participatory Practice* (GPP): *Guidelines for Biomedical HIV Prevention Trials, second edition (2011)* were developed to provide clinical trial sponsors and implementers with a formal stakeholder engagement framework. As one of the largest African research institutes, Wits Reproductive Health and HIV Institute (Wits RHI) became an early adopter of GPP by implementing its principles within large‐scale national and regional clinical trials. This article examines Wits RHI's lessons learned from implementing GPP, its ongoing efforts to institutionalize GPP, and the yet to be realized potential in creating fully sustainable structures for meaningful stakeholder engagement in HIV prevention research, implementation science and beyond.

**Discussion:**

For the past seven years, Wits RHI has undertaken both centralized leadership roles in implementing GPP across multi‐party regional research consortia as well as overseeing GPP for smaller investigator‐driven trials. Through this iterative roll‐out of GPP, key lessons have emerged. Obtaining upfront funding to support GPP activities throughout and between the research life cycle, and a trained multi‐disciplinary team of GPP practitioners have helped facilitate an enabling environment for GPP implementation. We further recommend formally integrating stakeholder engagement into study documents, including monitoring and evaluation plans with indicators and performance metrics, to assist teams to track and refine their GPP strategies. Finally, institutionalizing resources and supporting organization‐wide GPP along with ongoing support can help build efficiencies and maximize economies of scale toward a pragmatic and innovative application of the GPP Guidelines.

**Conclusions:**

Thanks to a growing global network of GPP practitioners and a burgeoning GPP Community of Practice, there has been substantive progress in making GPP an integral component of clinical HIV prevention research. The Wits RHI experience highlights the possibilities and the challenges to translating the GPP principles into concrete practices within specific clinical trials and across a research institute. Realizing the full potential of GPP, including direct and indirect – ‘collateral benefits’ will require the collective buy‐in and support from sponsors, implementers and community stakeholders across the research field. As the HIV prevention research field expands, however, a more conscious and systematic implementation of GPP is timely.

**Abbreviation**
CAB: community advisory board
CASPR: coalition to accelerate and support prevention research
CoE: centre of excellence
CRS: clinical research site
ECHO: evidence for contractive choices and HIV prevention options
EMPOWER: enhancing methods of prevention and options for women exposed to risk
FACTS: follow‐on African consortium for tenofovir studies
GCAG: Global Community Advisory Group
GCP: good clinical practice
GPP: good participatory practice
M&E: monitoring and evaluation
MOP: manual of procedures
MSM: men who have sex with men
NGO: Non‐Governmental Organization
PrEP: pre‐exposure prophylaxis
SOP: standard operating procedures
SRH: sexual and reproductive health
TAPS: Treatment and prevention for sex workers
TB: tuberculosis
UNAIDS: United Nations AIDS Programme
Wits RHI: wits reproductive health and HIV institute

## 1 Locating GPP within HIV prevention research

While clinical trials have long engaged with trial communities and other stakeholders as a matter of course, these efforts have often been *ad hoc*, unstructured and reactive. In the field of HIV prevention research, the need for a more systematic approach to community engagement became evident in the aftermath of the early and controversial closure of two oral pre‐exposure prophylaxis (PrEP) clinical trials in 2004 to 2005 1, 2, 3, 4. These premature closures in Cambodia and Cameroon highlighted the inherent power differentials within HIV biomedical clinical trials, and the complexity of undertaking effective stakeholder engagement in such settings. They also revealed how insufficient stakeholder engagement across the life cycle of a clinical trial may result in a number of damaging consequences. In this instance, the trial closures delayed clinical findings and subsequent product licensure, essentially derailing the product development and roll‐out timeline for PrEP as an additional tool that high‐risk individuals can employ to stay HIV‐uninfected.

In contrast to early AIDS treatment activism, which was led by people living with HIV and premised on the distinct “nothing about us without us” principle 5, 6, the constituency for HIV prevention activism is less well defined. The beneficiaries of biomedical HIV prevention trials include a diverse range of invested and affected individuals, from trial participants and civil society to governments and product developers. Even prospective end‐users vary widely. Some identify with high‐risk key populations, such as sex workers, men who have sex with men (MSM) or injection drug users, while others are at risk largely because of their geographical location and regional gender dynamics, such as women living in high prevalence communities. In short, while AIDS treatment activism was able to transcend these differences, no comparable over‐arching identity has yet formed to unify those in the field of HIV prevention. While trial participants remain at the centre of advocacy and engagement activities, there are diverse stakeholder groups and multiple partnerships involved, all of which exert varying degrees of influence in prevention trials.

The *Good Participatory Practice (GPP) Guidelines for Biomedical HIV Prevention Trials* developed by UNAIDS and AVAC in 2007 and revised in 2011 7 have provided a much‐needed, formalized framework to describe how clinical trial sponsors and implementers should engage with multiple stakeholders through deliberate, thoughtful and thorough mechanisms. The GPP Guidelines contribute to an overall body of normative guidelines and ethical goals of community engagement in research 8. At its core, GPP is premised on the same ethical principles of respect, beneficence, accountability and transparency that underlie Good Clinical Practice (GCP) 7, 9. While the primary focus of GCP falls on how clinical trials should be conducted with prescriptive guidance on the relationship between investigators and trial participants, GPP focuses more broadly on the relationships between *all* stakeholders in a trial 7. The GPP Guidelines offer a series of recommended steps for applying core principles, but few practical tools to guide stakeholder involvement in the often unpredictable social environment in which many HIV prevention trials are set. In this Commentary, we share the experiences of Wits RHI, a research institute at the University of the Witwatersrand, Johannesburg, South Africa, which became an early adopter and champion of GPP in HIV prevention research. Established in 1994, Wits RHI has a long history of community engagement, participatory research methods and working with local, national, regional and global partners 10, 11, 12. By leading GPP initiatives across multi‐site studies and regional research consortia, Wits RHI has been uniquely positioned to advance GPP implementation within research, and to develop models aimed at rendering GPP part of its institutional fabric.

In Follow‐on African Consortium for Tenofovir Studies (FACTS) 001, a phase III licensure trial of tenofovir 1% gel conducted at nine sites across South Africa 13, Wits RHI deliberately and systematically implemented the GPP Guidelines. Building on HIV prevention research studies’ community‐level stakeholder experience in the context of the GPP Guidelines 14, we reflect on how Wits RHI has applied GPP at local, national, regional and global levels. As the field evolves, Wits RHI continues to expand and adapt its original GPP tools in studies that have followed FACTS 001. Ultimately, Wits RHI is striving towards organization‐wide institutionalization of GPP, which will require re‐framing GPP beyond the scope and time frame of a single trial. In closing, we reflect on the ongoing challenges and advantages of embracing stakeholder engagement – which we characterize as “collateral benefits” – and the as yet unmet potential of GPP in HIV prevention research as a whole.

## 2 Developing a model to operationalize the GPP guidelines

**Figure 1:**
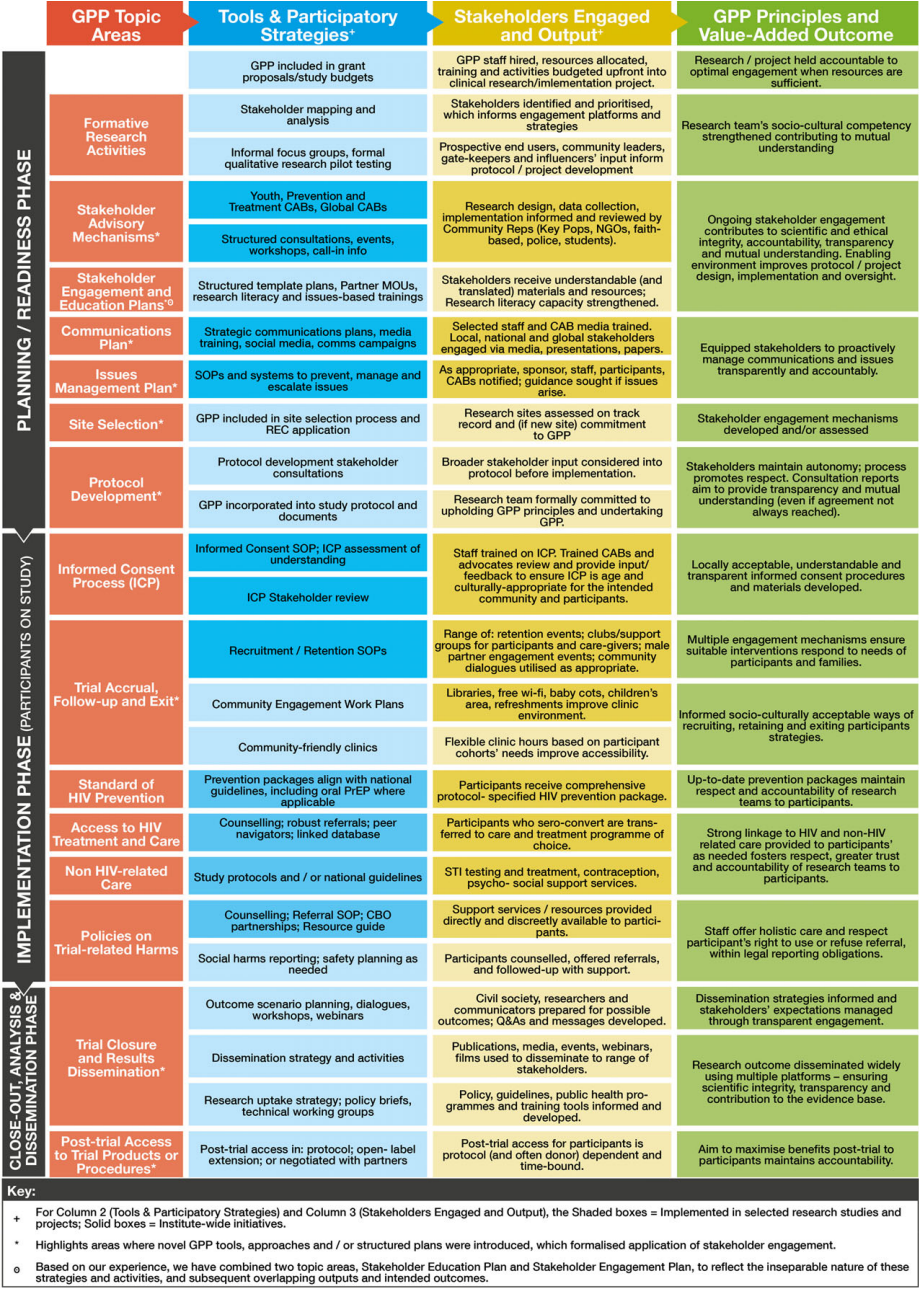
Wits RHI's Good Participatory Practice Implementation Model.

Launched in 2011, the same year as the revised GPP Guidelines, FACTS 001 was the first large HIV prevention trial to formally implement these guidelines. As the FACTS 001 Coordinating Team (led by Wits RHI) began to engage with the GPP Guideline's 16 topic areas that roughly follow the life cycle of a typical clinical trial 7, a rational clustering of these 16 areas into three manageable phases emerged. These phases comprised (1) study planning – including securing funds, developing protocol and study procedures, completing approvals processes, and securing study and site readiness; (2) implementing the study – including all time points in which study participants are actively being screened, enrolled or attending follow‐up visits; and (3) preparing for and disseminating study results – including data analysis, dissemination and research uptake, and policy influence work when applicable (See Figure 1). In addition to outlining a range of stakeholder engagement strategies, mechanisms, and tools, this model illustrates the value‐added outcome of attaining GPP – namely, the creation and sustaining of an enabling environment for research studies. While there is some overlap between the three phases, this division helps to facilitate the practical work of planning, resource allocation and oversight. How, then, has the model been implemented? And how has it has evolved since FACTS 001? In the remainder of this Commentary, we describe how the model has been applied beyond the traditional placebo‐control HIV prevention clinical trial context to open‐label studies, observational cohorts and implementation science studies at Wits RHI, each application contributing towards the larger goal of institutionalizing GPP. See Table 1 for a summary of referenced clinical trials and implementation studies with Wits RHI‐led GPP implementation.

**Table 1:**
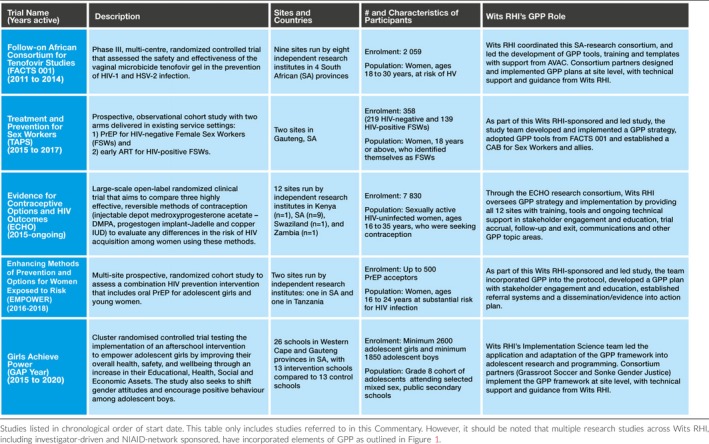
Clinical trials and studies with wits RHI‐led GPP implementation

## 3 Putting the GPP guidelines to work in Sub‐Saharan African clinical trials

Working in partnership with AVAC, the FACTS 001 Coordinating Team formally incorporated GPP from the early planning phase of the trial. A GPP section in the study‐wide Manual of Procedures (MOP) outlined the strategy, tactics and support that would be employed, and a series of novel tools were developed to support stakeholder engagement throughout the trial. Staff and community advisory board (CAB) trainings were convened before activation and during the study. A GPP site preparation checklist as well as structured templates for site‐specific plans (See Appendix S1 to S3) were developed to address stakeholder education, engagement, communications and issues management, which were regularly reviewed 15, 16. A study‐wide communications strategy that aligned with site‐specific GPP plans provided useful over‐arching guidance for internal and external stakeholder relationship management and rapid response situations 17. Even when site plans could not be implemented as designed, a framework was nevertheless in place to guide the adaptation of strategies to meet the evolving needs of the study. Oversight of implementation was provided by a full‐time GPP manager throughout the trial. It was this upfront investment by the FACTS 001 Coordinating Team, trial sites, and sponsors that would distinguish the study's formal approach to stakeholder engagement from traditional community engagement conducted during previous clinical trials.

As FACTS 001 drew to a close in mid‐2014, the process of preparing participants, trial communities and the broader research field for dissemination of study findings began. But this process was heavily influenced by parallel developments in the field. Around the same time, two other HIV prevention biomedical trials in women in Sub‐Saharan Africa, one of them which was stopped early, demonstrated lack of efficacy 18, 19. In some cases, blame for these outcomes was placed on the women study participants–accused of not adhering to the study products and then lying about this to trial staff 20, 21. Subsequent inquiry into these indications of sub‐optimal adherence seemed to confirm that indeed, there had been major discrepancies between self‐reported product use and pharmacokinetic measures of adherence 22. Nevertheless, the allegations that participants had lied brought to the surface deep‐rooted tensions associated with historic power disparities between marginalized, working class populations and an educated elite. These tensions continue to play out between community members, sponsors and implementers of HIV prevention trials in this region and beyond.

It was against this background that the FACTS 001 team began to prepare for study closure, primarily by actively strengthening study‐long stakeholder relationships. At each site, community dialogues were convened with local stakeholders to discuss possible outcomes and collectively consider how to communicate a study result to the trial site communities—regardless of the eventual findings. Similar consultations were convened with the study sponsors, national civil society leaders and HIV advocates. The AVAC‐led global Communications Working Group provided a further platform for coordinating messaging and outcome scenario‐planning efforts with communications officers of global, regional and national research groups. These efforts were aimed at managing expectations and – in the case that results would (and eventually did) show that tenofovir gel did not prevent HIV – pre‐emptively preparing to counter any sensationalized or inaccurate media coverage.

While the FACTS 001 study team implemented its GPP strategy across the three phases of study planning, implementation and results dissemination, unfortunately not all aspects of the GPP Guidelines could be applied, due in part to high staff turn‐over. This, in turn, resulted in the need for repeat training in GPP planning, execution and reporting. A further challenge emerged in finding ways to formally monitor the impact of the GPP activities without making reporting too burdensome 23.

Importantly, the implementation of GPP within this trial strengthened the capacity of these South African sites to engage more effectively with communities involved in research, as well as with other clinical trial stakeholders. Staff and CAB members at all nine sites were trained in GPP, and one site received technical support from Wits RHI to establish a new CAB altogether. Developing formal plans, such as an ‘issues management plan’, enabled even experienced sites to pro‐actively prepare for unexpected situations and hone their crisis de‐escalation skills. Seven of the eight research institutes involved in implementing the FACTS 001 trial were motivated to adopt at least some of the tools and practices learned during the trial, for use in other clinical studies and research programmes. In this way, FACTS 001 helped to set a new precedent for stakeholder engagement in HIV prevention research across multiple institutions in the country.

By the time FACTS 001 had concluded, GPP tools and training modules had become more readily available for use in trial settings globally 24, 25, 26, 27. Within the field of HIV prevention research specifically, GPP implementation had substantially expanded, and other research fields – from TB 28, 29 to emerging infectious diseases 30 – were joining the movement to absorb the 2011 guidelines into their approach. Building on our national‐level experience within FACTS 001, Wits RHI was tasked with leading the GPP and closely‐related Communications portfolios within the Evidence for Contraceptive Choices and HIV Prevention Options (ECHO) multi‐country open‐label randomized control trial. This trial is comparing three highly effective, reversible methods of contraception to evaluate differences in risk of HIV infection acquisition among women using these methods 31. ECHO has benefited from the adoption of existing tools and ability to repurpose activities, such as community dialogues, that proved to be beneficial to FACTS 001. Investigators on ECHO have also incorporated GPP and a number of related recommendations directly into the study protocol and MOP. During the planning phase, stakeholder engagement criteria were added to the site selection process and a GPP expert joined the site selection committee. Once sites had been chosen, they developed GPP plans and began engagement activities, which were documented in a GPP site activation checklist.

For five of the 12 sites, including two sites in South Africa and one each in Kenya, Swaziland and Zambia, ECHO's implementation of GPP has been a novel experience. However, within this cohort, two sites were new to running clinical trials altogether while the other three sites were seasoned in community engagement but new to applying a formal process. In addition to the traditional site‐based CABs that include local community leaders and constituency representatives, the study has further established a Global Community Advisory Group (GCAG). Bringing together advocates and other civil society stakeholders that work at the intersection of reproductive health and HIV prevention at national, regional and international levels, this additional engagement mechanism has provided a platform for invested individuals and coalitions that operate outside of specific trial countries to engage directly with the research team. GCAG members review study documents, such as the informed consent forms, participate in quarterly calls with the study leadership, and where feasible, engage with staff at research sites and local CAB members through site visits. Funding for these activities remains a challenge, with advocates and others volunteering their time to serve as GCAG members.

### 3.1 Institutionalizing GPP

As these individual clinical trials embraced GPP, Wits RHI began to expand its efforts to build African expertise in applying GPP principles and adapting its recommendations more widely within the institute. A small band of three to five staff led most of this work, and began training entire multi‐disciplinary teams working outside of the scope of clinical research – such as implementation science programmes charged with providing technical support to local public health clinics. With each training, we assessed how each of the 16 topic areas were relevant or could be adapted, and identified opportunities to collaborate between projects and noted gaps in stakeholder relations management. It quickly became evident that many projects had overlapping stakeholders, yet there was little optimal coordination of their engagement activities. At the request of senior leadership, Wits RHI established a committee to streamline community outreach and recruitment activities in 2016. The aim of the institute‐wide committee was to identify and leverage synergies between projects and coordinate stakeholder relationship engagement and research participation across geographical areas, facilities and cohorts 32. With representatives from across the institute's research, technical assistance and service provider portfolios, this unfunded group created a platform to integrate the GPP principles within institutional practices, develop shared resources, and regularly assess stakeholder partnerships and mechanisms for engagement. For example, through a series of compulsory workshops, over 110 community and outreach staff at Wits RHI have been trained on the practical application of GPP principles and tangible ways to strengthen coordination and referrals between research studies on the one hand, and health and social services on the other. The working group has spearheaded efforts to launch an ethics‐approved, locally‐focused social media campaign, and to coordinate participation in community radio shows that promote community stakeholder education. Job aids are being developed to assist staff in explaining the different research studies to prospective participants, with the aim of empowering them to choose the research or health service that best meets their needs and interests.

These efforts have been bolstered by a USAID‐funded and AVAC‐led grant, the Coalition to Accelerate and Support Prevention Research (CASPR), which is supporting Wits RHI to extend GPP across the institute with the ultimate aim of establishing a GPP Centre of Excellence (CoE) – a global first. While CoEs are traditionally characterized as “physical or virtual centres of research which concentrate existing capacity and resources to enable researchers to collaborate across disciplines and institutions on long‐term projects that are locally relevant and internationally competitive in order to enhance the pursuit of research excellence and capacity development” 33, in the case of GPP – it is precisely these research outputs and capacity development initiatives that are currently lacking and in need of substantive investment if the field as a whole is to effectively put these principles into practice.

Within the context of a growing and global GPP Community of Practice, the aspirational aims of a GPP Centre of Excellence are thus: 1) to build internal cohesion and capacity to implement standardized, yet dynamic GPP tools and strategies across diverse research studies and projects within Wits RHI; 2) document practices and share GPP resources beyond the institute; and 3) nurture the development of GPP specialists across disciplines, deepening their ability to critically analyse and evaluate the true costs, benefits and potential impact of utilizing GPP within HIV prevention and related research. For example, Wits RHI has developed a GPP Leadership Program that trains course participants from around the world on the often overlooked skills of how to negotiate budgets, develop M&E indicators 8, 27, gain institutional buy‐in, and engage strategically with reluctant stakeholders, among other critical leadership skills for GPP. Learning to navigate these multi‐layered and often messy terrains is integral to building the next generation of GPP champions needed to advance implementation beyond the small cohort of enthusiasts currently leading the field.

### 3.2 Lessons learned

As Wits RHI continues to invest in institutionalizing GPP, and the dividends begin to materialize, a series of key lessons have emerged. First, throughout the GPP Guidelines, it is stipulated that “trial sponsors [should] ensure sufficient funding and research teams [should] create a budget and allocate funds and staff time” 7 to cover GPP activities. In practice, we have found that this is best achieved via an early commitment to include GPP‐dedicated human resources, activities and oversight mechanisms into the initial grant and budget proposal. While donors increasingly request line‐item budgets for recruitment and retention activities, CAB engagement and dissemination plans, limiting GPP to these items alone leaves little room for sustaining ongoing relationships or designing innovative approaches. From developing social media campaigns beyond the scope of a specific trial to supporting relevant broad‐based initiatives, these activities can prove cost‐saving in the long run. Strategies that may appear superlative on the surface can save time and money by building a research‐informed and empowered network of stakeholders that can effectively engage without research teams having to “start from scratch” every time a new study enters the research field.

In addition to funding GPP activities within study‐specific life cycles, we have found it is the time periods between close‐out of one study (and its related contract) and the start of the next, where ongoing communication with stakeholders is still required, and yet often neglected 34. Even as a large institute and pluri‐potent site running multiple clinical trials and research studies, minimizing time lapses between stakeholder activities requires resourcefulness and cost‐sharing.

Second, it takes a team to make GPP work. Without the vested buy‐in from trial sponsors, lead investigators and the full research team, endeavours left solely to community and outreach staff are often hamstrung. When lead investigators and clinicians participate in consultations and community dialogues, myths can be debunked, clinical procedures can be clarified and ethical issues can be examined as researchers and community members grapple to resolve research design and implementation challenges together. Not all recommendations are adopted, but the indirect benefits of broaching open and tough conversations builds trust even amidst disagreement.

Even more, rolling out GPP across the Institute has led *us* to continually re‐define the parameters of who is included in this “us” as we strive to uphold the “Nothing about us without us” adage. Wits RHI maintains three independent CABs, including Youth, Prevention and Treatment focused CABs. Until recently, we also convened a distinct CAB for Sex Workers and community members who work closely with them. While these mechanisms support stakeholder autonomy, our research studies recruiting key populations also benefit from and are better equipped to uphold GPP principles of respect, mutual understanding and accountability by employing peer navigators and educators that self‐identify with the communities being served – whether adolescents, sex workers or MSM. As we move towards over‐arching strategies, shared approaches and efforts to combine engagement activities, we are working to find the right fine balance between meeting the specific needs of distinct populations while maximizing efficiencies with limited resources.

Third, the more ways GPP is integrated into formal trial documentation and procedures, the more likely it is to be successfully implemented. This holds true because trial budgets generally align with what is in the protocol, which in turn is monitored. For instance, in the ECHO Study, the Management Committee regularly reports progress on the study's GPP activities to the donors and to the Data and Safety Monitoring Board. Likewise, staff are trained to follow the study‐specific operating procedures – processes that are no longer optional add‐ons, but rather deemed integral to study success.

Related to this, more could be done to strengthen monitoring and evaluation (M&E) of GPP strategies and activities in studies. Currently, most indicators focus on quantifiable outputs, such as CAB meeting attendance or number of workshops convened. Additional metrics could be developed to measure both the quality of community and stakeholder engagement outcomes, and the impact it spurs. This is particularly important, lest one succumb to the notion that meaningful engagement is subjective and difficult to measure. Taking an evidence‐based approach, the GPP team at Wits RHI is working to identify lead (input oriented) and lag (outputs) indicators, so that the impact of GPP activities may be better assessed, refined and evaluated. While lag indicators are relatively easy to measure, they can be difficult to improve or influence. Leading indicators, by contrast, are characteristically harder to measure but easy to influence 35. As a result, standard M&E plans tend to only include lag indicators. However, it is the hard‐to‐measure lead indicators (e.g. rumours in the community, research literacy levels, clinic accessibility) that often determine and influence lag indicators (e.g. study‐specific recruitment and retention rates, or number of people who attend stakeholder engagement activities). While many GPP‐related lead indicators can be initially uncovered via “ear to the ground” tactics used by CABs and by outreach staff working directly in communities, we should not underestimate the use of qualitative research methods to generate empirical data and elucidate underlying patterns in people's experiences of engagement. Possible methods could include formal pre‐ and post‐workshop evaluations, focus group discussions and waiting room observations conducted by trained social scientists.

A final lesson learned relates to sustainability: there are critical roles for technical assistance provision, education and training, and information and resource sharing within and between teams, especially when it comes to building institution‐wide GPP platforms that can outlast staff changes and organizational upheavals. Maintaining knowledge management systems and securing institutional memory are often lacking in non‐profit organizations. This is a particular danger in the nascent field of GPP, where there is an overall lack of documented evidence 34. To address this, Wits RHI has set up a resource repository within its internal intranet to strengthen coordination, build efficiencies and embed GPP within the institute. Highlighting specific ways that investing in GPP tools improve donor‐monitored performance metrics, providing guidance on how to add GPP‐related Key Performance Areas to job descriptions, and regularly discussing how teams can “live” the organizational values and GPP principles are all lending to embedding Wits RHI with a structured and formal approach to stakeholder engagement as outlined in the GPP Guidelines.

## 4 Conclusion

**Figure 2:**
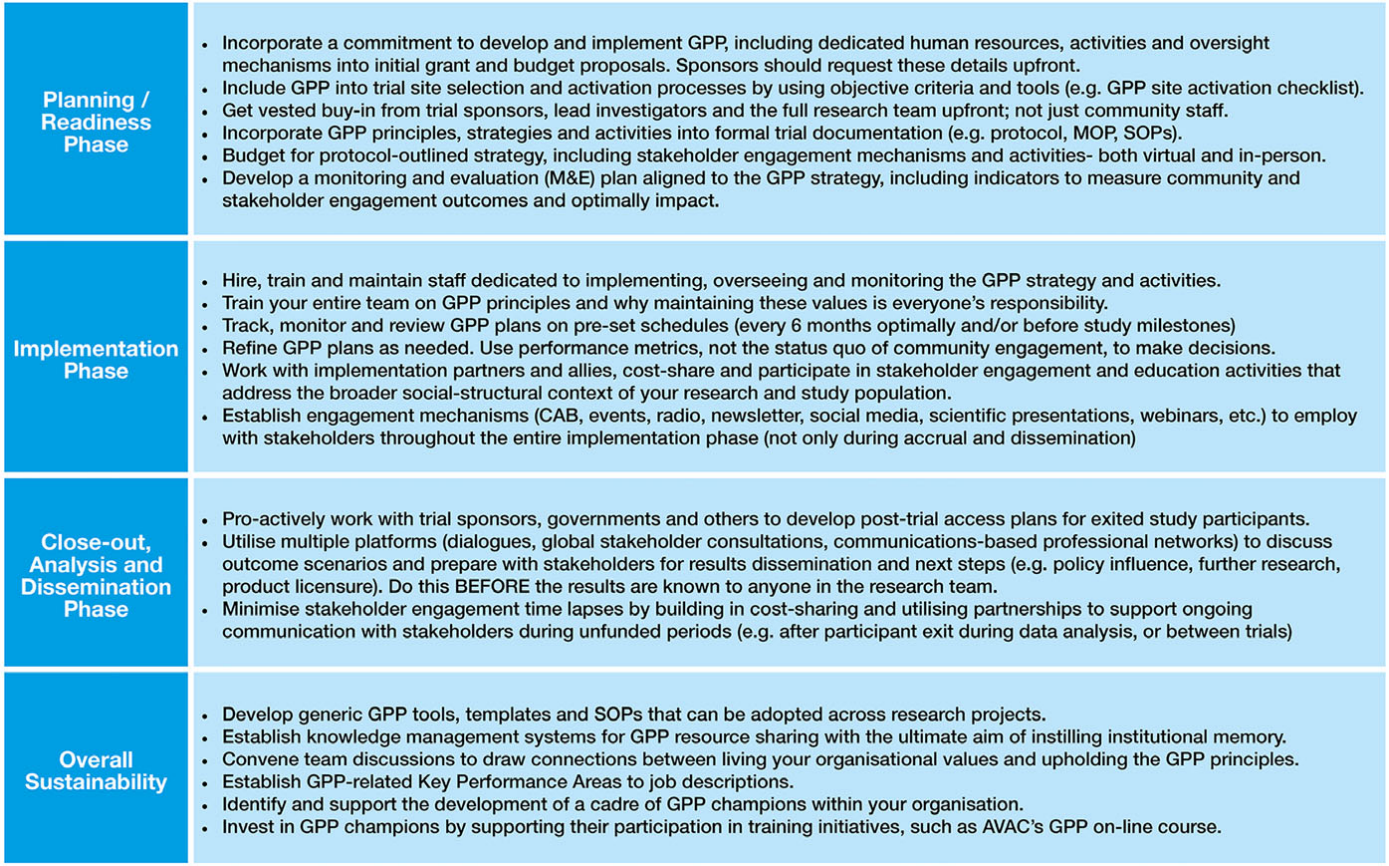
Key Recommendations for Implementing Effective and Sustainable Good Participatory Practice.

Over the past decade, there has been tremendous progress in making GPP an integral component of clinical HIV prevention research. A plethora of practical tools and an introductory on‐line training course have been developed 24, 25, 26, 27, and the concept of “stakeholder engagement” is increasingly part of the lexicon of clinical research. Our experiences and lessons learned illustrate that a number of challenges remain before the full potential of GPP may be realized. Still, through existing efforts, there are achievable recommendations that research institutes, sponsors and implementation partners committed to GPP can undertake. These are outlined in Figure 2.

Beyond the visible and often cited benefits of GPP, such as improved participant retention and decreased rumours in the community, it is the strengthened relationships and intangible trust that meaningful engagement fosters. From there, shared visions and partnerships for ethical and much‐needed research studies can flourish. We have deemed these outcomes based on lived principles as ‘collateral benefits’, those that accrue from not merely implementing, but also from re‐imagining GPP. As HIV prevention clinical trial design becomes ever more complicated, and biomedical research itself expands – with an estimated 25,000 trial participants now enrolled in research studies globally 36 – it has never been more a prudent time to invest in GPP.

## Competing interests

The authors do not have any conflicts of interest to declare.

## Authors’ contributions

DB drafted the first draft of the manuscript, developed the conceptual model with input from co‐authors and managed revisions. DB, TE, MM, NM, SP and SDM discussed key ideas and concepts forming the basis of this commentary. FS and SDM reviewed and substantively revised early drafts. All co‐authors reviewed and gave input on the final draft, and cleared it for submission.

## Supporting information


**Appendix S1.** FACTS 001 GPP Site Preparation Plan (2011).

**Appendix S2**. Revised Wits RHI's GPP site preparation and activation checklist (2017).

**Appendix S3**. GPP strategic plan template.
