# Supplementary material for: Collateral benefits: how the practical application of Good Participatory Practice can strengthen HIV research in sub‐Saharan Africa
Source: J Int AIDS Soc. 2018 Oct 18;21(Suppl Suppl 7):e25175. doi: 10.1002/jia2.25175 (PMC6193316; doi:10.1002/jia2.25175)
Supplement: Supplementary file 1 — Appendix S1. FACTS 001 GPP Site Preparation Plan (2011). [file JIA2-21-e25175-s001.docx]

| **Activity** | | **Person**  **responsible** | | **Estimated date of completion** | **Tick when completed** | **Comments on status of activity** |
| --- | --- | --- | --- | --- | --- | --- |
| Create list of key informants and relative stakeholders that may assist with planning, implementation and reviewing process | |  | |  |  |  |
| Create database of all local stakeholders for community activities (including all NGO, CBO, etc.) | |  | |  |  |  |
| Form a site Community Advisory Board for FACTS 001 (if CAB exists across other research projects this does not apply) | |  | |  |  |  |
| Identify other Community Advisory Mechanisms which will be used during FACTS 001 (e.g. community radio, community events, etc.) | |  | |  |  |  |
| Create a budget and allocate sufficient funds to support ongoing stakeholder advisory mechanisms | |  | |  |  |  |
| Draft a stakeholder engagement plan which describes strategies and mechanisms that will be used to engage with a broad range of stakeholders | |  | |  |  |  |
| Create a detailed site specific stakeholder education plan which should describe strategies and mechanisms for providing relevant education. | |  | |  |  |  |
| Map out a detailed site specific communications plan that describes information to be communicated externally at various stages of the research life-cycle. | |  | |  |  | Core to develop SOP for all sites (Media Communications & Crisis Management). |
| Create a site specific issues management plan to describe how the research team will manage issues or unexpected developments in the FACTS001 trial | |  | |  |  | Core to develop Media Communications & Crises Management SOP for all sites. |
|  | | | | | | |
| Checklist completed by: | Signature: | | Date: | | |  |
|  |  | |  | | |  |
| FACTS PI/ Organization Director: | Signature: | | Date: | | |  |
|  |  | |  | | |  |

**Appendix 1: FACTS 001 GPP Site Preparation Plan (2011)**
