# Supplementary material for: Collateral benefits: how the practical application of Good Participatory Practice can strengthen HIV research in sub‐Saharan Africa
Source: J Int AIDS Soc. 2018 Oct 18;21(Suppl Suppl 7):e25175. doi: 10.1002/jia2.25175 (PMC6193316; doi:10.1002/jia2.25175)
Supplement: Supplementary file 2 — Appendix S2. Revised Wits RHI's GPP site preparation and activation checklist (2017). [file JIA2-21-e25175-s002.docx]

Appendix 2: Revised Wits RHI’s GPP Site Preparation and Activation Checklist (2017)

| **Study name:** | **Expected start date for accrual:** |
| --- | --- |
| **Date (last date updated):** | **Anticipated sample size to enroll:** |

| **Activity** | | **Tool/Resource to submit** | | **Person**  **responsible** | **Date Completed** (note “pending” if not completed) | | **Comments on status** |
| --- | --- | --- | --- | --- | --- | --- | --- |
| **Conduct stakeholder mapping exercise** of local stakeholders for community education, outreach activities and any referral services to be used during study | | *Stakeholder mapping excel* | |  |  | |  |
| **Develop a site-level GPP strategic plan***, including: Stakeholder engagement plan, Recruitment and Retention plan, Site-specific communications and issues management plan | | *GPP Plan Template* | |  |  | |  |
| **Develop / Adapt SOPs or Work Instructions on outreach, recruitment and retention** | | *Generic SOPs* | |  |  | |  |
| **Develop budget with input from IoR, Study Coordinator and Community Team** (Aim is to allocate sufficient funds for stakeholder engagement activities outlined in approved GPP plan (i.e. community education, recruitment, retention, dissemination) | | *Site budget* | |  |  | |  |
| **Community education materials –** Develop educational and outreach materials required for study (e.g. slide deck, brochure, pamphlet, film). Vet draft materials with CAB | |  | |  |  | |  |
| **Community Advisory Board** – Investigator of Record (IoR) or designee to present study protocol and ICFs to CABs. Collate questions/feedback for protocol team’ review. (NB: Focus on CABs with scope relevant to specific study but please present to all CABs) | | *Slide deck for community level audience* | |  |  | |  |
| **Conduct outreach and information sessions** with local stakeholders outlined in stakeholder list BEFORE the study begins. These should include key gatekeeper and local leaders who are essential to facilitating a successful implementation of the study. | |  | |  |  | |  |
| **Set up referral systems** – Set up formal referral systems for study participants who may require services outside of institute’s scope (e.g. rape counselling; legal services; shelters) | |  | |  |  | |  |
| Checklist completed by: | Role in Study: | | Signature: | | | Date: | |
|  |  | |  | | |  | |
| Reviewed by (IoR/ Study Coordinator): | Role in Study: | | Signature: | | | Date: | |
|  |  | |  | | |  | |

*RHI template is available. If Study Network or sponsor provides template, please cross-reference to ensure all areas covered. GPP Plan may be developed by the Community Team, but should have sufficient input from the IoR, Study Coordinator and be presented at least once to the overall Study Team. Plan should also be reviewed with the primary consultative CAB before being finalised.
