# Supplementary material for: Collateral benefits: how the practical application of Good Participatory Practice can strengthen HIV research in sub‐Saharan Africa
Source: J Int AIDS Soc. 2018 Oct 18;21(Suppl Suppl 7):e25175. doi: 10.1002/jia2.25175 (PMC6193316; doi:10.1002/jia2.25175)
Supplement: Supplementary file 3 — Appendix S3. GPP strategic plan template. [file JIA2-21-e25175-s003.docx]

**[INSERT PROJECT LOGO IF RELEVANT]**

**[INSERT PROJECT NAME]**

**Good Participatory Practices**

**Strategic Plan**

**Project Name:**

**Location / Site / Scope:**

**Submitted by:**

**Approved by:**

**Date:**

**Date Submitted:**

Contents

[SECTION 1: Stakeholder Engagement Objectives 3](#_Toc448441682)

[SECTION 2: Formative Research / Landscape Analysis 3](#_Toc448441683)

[SECTION 3: Community & Stakeholder Analysis 5](#_Toc448441684)

[SECTION 4: Communications and Issues Management Plan 6](#_Toc448441685)

[SECTION 5: Participant Recruitment (for projects that recruit clients or study participants) 8](#_Toc448441686)

[SECTION 6: Participant Retention 9](#_Toc448441687)

[SECTION 7: Findings / Results Dissemination Plan 10](#_Toc448441688)

# SECTION 1: Stakeholder Engagement Objectives

*Instructions to be deleted once section completed: Please list at least 3 objectives for your stakeholder engagement strategy. These objectives will inform the activities and other plans outlined throughout this strategy. I.e. Each section below should trace back to these objectives.*

*Over the course of the study/project life cycle, the priority of the objectives may shift, as might the dynamics between stakeholders, and the relationship of key stakeholders to the project. Please include objectives that address the phases of the project (1) for research, these include: activation, recruitment, retention, study close-out. 2) For implementation projects, these phases may include start-up and planning, implementation, handover /close-out. Public perception and support, stakeholder engagement, working with partners, and dissemination of findings are important for all types of projects.*

| **Objective 1** |
| --- |
|  |
| **Objective 2** |
|  |
| **Objective 3** |
|  |

# SECTION 2: Formative Research / Landscape Analysis

*Instructions to be deleted once section completed: The purpose of this section is to gauge the level of knowledge, experience and preparedness amongst your team and populations that will be involved in, affected by, or have influence over the project, which will in turn, help you plan your activities accordingly.*

*Please make this section relevant to your project. For example, you may list formative research activities that you have done in the past (e.g. community or cognitive mapping), or cite other organisation’s work that will inform this project. (Reference: pg. 27-28 2011 GPP Guidelines)*

| **Write a brief description of your geographic site location (e.g. population, HIV prevalence, key health issues) and the project team’s experience at this location (is this a new area for RHI? Or building on established partnerships?)** | |
| --- | --- |
|  |  |

**THIS NEXT SECTION IS FOR RESEARCH STUDIES (OR OTHER PROJECTS) THAT WILL ACTIVELY RECRUIT PARTICIPANTS/CLIENTS (PLEASE SKIP IF NOT RELEVANT TO YOUR PROJECT)**

| **Sociocultural landscape** |
| --- |
| 1) Please list (you can use bullet points) attitudes, beliefs and sociobehavioural factors among your **potential trial population** that could interfere with recruitment, retention or trial conduct (e.g, misconceptions about research, mistrust of research products, social stigma, religious or traditional belief or practices, gender discrimination). |
| 2) Please list attitudes, belief and socio behavioral factors among key gate-keepers (e.g. for studies recruiting young women, this could include their male partners and parents) of potential trial participants in your community that could interfere with recruitment, retention or trial conduct (e.g. misconceptions about research products, belief that parents or partners should approve of trial participation, mistrust in research, etc.) |
| 3) Please list any additional attitudes, belief, or sociobehavioral factors not already listed that may arise from the **local site community, including local media and the population around your trial SITE THAT** could interfere with recruitment, retention or trial conduct. |

# SECTION 3: Community & Stakeholder Analysis

*Please outline the stakeholders to be engaged for your project, and specify the activities and mechanisms you plan to use. Please ensure to include relevant non-governmental organisations and community-based organisations. You may also want to think about and include what level of engagement is required for each stakeholder (e.g. consultation, collaboration, etc.). This list is not meant to be exhaustive; it is meant to be strategic.*

*Recommendation: Please transfer this spreadsheet into Excel for easier updating and maintenance.*

| **Key Stakeholders Analysis** | | | | | |
| --- | --- | --- | --- | --- | --- |
| **Stakeholder group and brief description** (e.g. Local DOH, women’s group, NGOs, clinic) | **Contact person name, job title and email** (List main point of contact at organisation*) | **Physical address, including postal code** (This will inform our GPS geo-spatial mapping of stakeholders, so completeness is key) | **Area of expertise/ influence** (e.g. policy, implementer, gate-keeper) | **Objective / Level of engagement / Existing relationship** (e.g. partner to seek input/buy-in, assist with recruitment effort, info-sharing only, etc.) | **Engagement mechanism** (Refer to table below) |
| *Example: Youth unite, civil society group that advocates for expanding method mix in clinics* | *Sue Supporter, Director sue@supporter.co.za* | *Corner of Klein and Pata street,*  *Hillbrow 2192* | *High-profile advocate and influencer in national policy circles (SANAC)* | *Partner / Hope they can assist with outreach and education efforts through their networks.*  *Currently, RHI refers young people to get IUDs at their clinics* | *Will invite Sue and her group to all events. Given Sue is senior, we always invite her project manager also.* |
| *Example: Referral clinic* | *Samantha Sceptic, Clinic Manager, no email* | *33 Juta Street*  *Braamfontein*  *2291* | *Gate keeper and influencer* | *Information-sharing with aim of making them ally*  *Currently, we refer complicated HIV-positive cases to them – but often don’t get feedback on cases* | *One-on-one briefings/ In-person meetings.*  *Send newsletters.* |
| ADD ROWS AS NEEDED – DELETE GREEN EXAMPLES |  |  |  |  |  |

# SECTION 4: Communications and Issues Management Plan

*Please use template below to outline the communications point persons and processes for maintaining open communications and meeting the information needs of local stakeholders.*

**Section A: Internal Communication**

**Part 1: Internal Project Contacts**

| **Name** | **Job Title / role** | **Email** | **Work extension AND mobile number** | **Allowed to speak with media? Yes/No** |
| --- | --- | --- | --- | --- |
|  |  |  |  |  |
|  |  |  |  |  |
|  |  |  |  |  |
|  |  |  |  |  |
|  |  |  |  |  |

**Part 2: Project Team Meetings:**

| **Frequency of team meetings  (e.g. monthly, weekly, etc.)** |  |
| --- | --- |
| **Date/ time/ location of site team meetings** | Day of week:  Time:  Location: |
| **People expected to attend team meetings** *(List all)* | |
| **Please describe system for documenting team meetings:** (Does someone keep minutes? Where are the minutes stored? Are they accessible to all staff?) | |

**Section B: Issues Management** *While there are many known issues that could emerge and undermine the success of a project, this strategy assists studies and projects to develop a process for managing the unexpected developments and emerging concerns (e.g. fire on site; riots in area; sexual abuse charge involving staff, etc.)*

*Having this process in place before an issue arises prepares the site to respond swiftly and responsibly if an issue does arise.*

| 1. **Who are the key project / study staff who are responsible for addressing emerging issues?** |
| --- |
| 1. **Please outline the chain of communication within the team and with relevant stakeholders for emerging issues (E.g. Who is responsible for notifying Senior Leadership within RHI?, project partners? Local DOH if needed? etc.).** |
| 1. **How does your project document / record issues that emerge, how they are responded to and their outcome?** |

**Section C: Media Communication**

| **Project Media Contacts** | | |
| --- | --- | --- |
|  | **Name (Position/ title)** | **Contact Details** |
| **Primary Media Point Person** |  |  |
| **Back-up** |  |  |
| **Additional Spokespersons** |  |  |

| **Please describe the process when media queries are received (e.g. what happens when a journalist calls or comes to the site/project?):** |
| --- |

# SECTION 5: Participant Recruitment (for projects that recruit clients or study participants)

*Please outline the recruitment strategies your site is planning to utilise during the study/project. Please refer to any study-specific guidance, recruitment SOPs or language from HREC applications, as applicable.*

| 1. **Briefly describe your catchment area, including specific geographical locations where recruitment activities will take place.** |
| --- |

| 1. **Outline Specific Locations and Recruitment Activities** | | |
| --- | --- | --- |
| **Recruitment Locations** (STI, family planning, VCT clinics, etc.) | **Activities Strategies** (Distribute materials, Talks, Referral System, Events) | **Person/s Responsible** |
|  |  |  |
|  |  |  |
|  |  |  |

| 1. **List any other specific outreach strategies your site will use to recruit high-risk women, including adolescents/Young women** (E.g. Social media, peer Educators, Flash Mobs, Community Theatre, Etc.) |
| --- |

| 1. **List any pre-screening activities your site is planning** (E.g. any educational session, checklist or activity to assess interest and eligibility before women undergo the Screening visit ICF) |
| --- |

| 1. **List anticipated challenges and how the site is pro-actively planning for these.** |
| --- |

# SECTION 6: Participant Retention (for projects that recruit clients or study participants)

*Please outline the recruitment strategies your site is planning to utilise during the study/project. Please refer to any study-specific guidance, SOPs or language from HREC applications, as applicable.*

| 1. **Outline your site/project procedures for visit reminders and participant/client follow-ups when there is a missed visit** (Feel free to cut and paste from SOP). |
| --- |

| 1. **List site and community-level activities and strategies planned to maintain high retention** (E.g. Evening or Weekend clinic hours**,** Events) |
| --- |

| 1. **List anticipated challenges and how the site is pro-actively planning for these.** |
| --- |

# SECTION 7: Findings / Results Dissemination Plan

*Please include a brief description of your results dissemination plan. Implementation and service delivery projects, especially those that include an evaluation, should also plan to disseminate findings. This plan should include:*

- *Methods and strategies for results dissemination for research studies (e.g. participant meetings, bulk SMS, CAB/stakeholder meetings, media outreach);*
- *List of all stakeholders to whom you will disseminate the results and which method or strategy you will use;*
- *Adequate budget amount for carrying out of strategies.*

*(Reference: pg. 60-63 2011 GPP Guideline)*
